# ﻿*Meotipa* species (Araneae, Theridiidae) from China

**DOI:** 10.3897/zookeys.1082.75400

**Published:** 2022-01-20

**Authors:** Zhongwei Deng, Ingi Agnarsson, Zhanqi Chen, Jie Liu

**Affiliations:** 1 CAS Key Laboratory of Tropical Forest Ecology, Xishuangbanna Tropical Botanical Garden, Chinese Academy of Sciences, Mengla, Yunnan 666303, China; 2 Hubei Key Laboratory of Regional Development and Environmental Response, Faculty of Resources and Environmental Science, Hubei University, Wuhan 430062, China; 3 The State Key Laboratory of Biocatalysis and Enzyme Engineering of China, College of Life Sciences, Hubei University, Wuhan 430062, Hubei, China; 4 University of Vermont, Department of Biology, 109 Carrigan Drive, Burlington, VT 05405–0086, USA; 5 School of Nuclear Technology and Chemistry and Biology, Hubei University of Science and Technology, Xianning 437100, Hubei, China

**Keywords:** Cobweb spider, comb-footed spider, flattened black spines, long-legged theridiid spiders, new species, taxonomy

## Abstract

In an ongoing effort to expand knowledge of the Chinese cobweb spider fauna (Theridiidae), the genus *Meotipa* Simon, 1894 is reviewed. Two new species are described, *Meotipapseudopicturata***sp. nov.**, *Meotipastriata***sp. nov.**, and five known species are redescribed: *Meotipaargyrodiformis* (Yaginuma, 1952), *Meotipapulcherrima* (Mello-Leitão, 1917), *Meotipapicturata* Simon, 1895, *Meotipaspiniventris* (O. Pickard-Cambridge, 1869), and *Meotipavesiculosa* Simon, 1895.

## ﻿Introduction

*Meotipa* Simon, 1895 is an enigmatic and taxonomically poorly understood theridiid genus, in part because it was synonymized with the much larger and more widespread genus *Chrysso* by Levi (1962) until being resurrected by Deeleman-Reinhold (2009). *Meotipa* are long-legged theridiid spiders bearing a conspicuous synapomorphic feature: flattened black spines on the abdomen and/or legs (Deeleman-Reinhold 2009). However, some *Chrysso* species also have obvious spines on the abdomen which are often sharp or needle-like (Kulkarni et al. 2017), such as *Chryssolingchuanensis* Zhu & Zhang, 1992 known from China. Their relationship needs further research based on more evidence, such as molecular data. *Meotipa* contains 18 known species (World Spider Catalog 2021) that are quite common in gardens and secondary forests across the tropics in Asia (Deeleman-Reinhold 2009; Kulkarni et al. 2017). Nine *Meotipa* species are known from China: *M.argyrodiformis* (Yaginuma, 1952), *Meotipacapacifaba* Li, Liu, Xu & Yin, 2020, *Meotipaluoqiae* Lin & Li, 2021, *Meotipamenglun* Lin & Li, 2021, *M.picturata* Simon, 1895, *M.pulcherrima* (Mello-Leitão, 1917), *M.spiniventris* (O. Pickard-Cambridge, 1869), *M.vesiculosa* Simon, 1895, and *Meotipazhengguoi* Lin & Li, 2021.

In the past two years, a series of surveys for Chinese theridiid spiders were conducted by the colleagues of Hubei University in China and yielded numerous new species. This is our second paper on Chinese cobweb spiders with the aim to review the Chinese *Meotipa* spiders and includes two new species, one new record for Hunan, and four known species (Li et al. 2021).

## ﻿Materials and methods

In the field, we collected cobweb spiders by using visual searching and beating vegetation. We attempted to take photographs of every species, alive, in the field, and webs of all species encountered in web were photographed. All specimens were preserved in 99% ethanol and examined with an Olympus SZX16 stereomicroscope; details were further investigated with an Olympus BX51 compound microscope. Male palps and female genitalia were examined and their photographs taken after dissection from the spider bodies. The epigynum was cleared with Proteinase K. Habitus and photographs were obtained using a Leica 205C digital microscope. We added some key marginal lines for genitalia photographs using the Apple pencil (2^nd^ generation) and edited the photographs in Adobe Photoshop 2020. Leg measurements are shown as: total length (femur, patella, tibia, metatarsus, tarsus). The terminology used in text and figure legends, and palpal homologies follow Agnarsson (2004a) and Agnarsson et al. (2007a). Geographical co-ordinates were recorded in decimal degrees. All measurements are given in millimeters. All specimens are deposited in the Centre for Behavioural Ecology and Evolution, College of Life Sciences, Hubei University, Wuhan, China (**CBEE**). The abbreviations used in this paper are as follows:

**ALE** anterior lateral eyes;

**AME** anterior median eyes;

**C** conductor;

**CD** copulatory duct;

**CO** copulatory opening;

**E** embolus;

**FD** fertilization duct;

**MA** median apophysis;

**PE** posterior eyes;

**PLE** posterior lateral eyes;

**PME** posterior median eyes;

**S** spermathecae;

**SE** stuck emboli.

**ST** subtegulum;

**T** tegulum;

## ﻿Taxonomic account

**Family Theridiidae Sundevall, 1833**

### ﻿Subfamily Theridiinae Sundevall, 1833

#### ﻿Genus Meotipa

Taxon classificationAnimaliaAraneaeTheridiidae

Simon, 1895

BD69515D-D6D7-5229-BEE0-05832F322318

Table 1

##### Diagnosis.

Female of *Meotipa* differs from all other theridiids by the conspicuous flattened black spines on the abdomen and/or legs. Therefore, they are often referred to as spiny theridiids. *Meotipa* males are significantly smaller than females. The male palp of *Meotipa* can be distinguished from other theridiids by the spoon-shaped conductor and embolus being thin and short, with the straight base, completely surrounded by the spoon-shaped conductor or the tip of embolus, which is opposite to the conductor (except *Meotipabituberculata*, see Deeleman-Reinhold 2009: 419, figs 20–37). The species included in *Meotipa* are given below, with their distributions.

**Table 1.** The distributions of known *Meotipa* species in the world.

*Meotipaandamanensis* (Tikader, 1977) (♀)	India (Andaman Is.)
*Meotipaargyrodiformis* (Yaginuma, 1952) (♂♀)	China, Japan, Philippines, India
*Meotipabituberculata* Deeleman-Reinhold, 2009 (♂♀)	Indonesia
*Meotipacapacifaba* Li, Liu, Xu & Yin, 2020 (♂♀)	China
*Meotipaimpatiens* Deeleman-Reinhold, 2009 (♂♀)	Malaysia, Indonesia
*Meotipaluoqiae* Lin & Li, 2021 (♂)	China
*Meotipamakiling* (Barrion-Dupo & Barrion, 2015) (♀)	Philippines
*Meotipamenglun* Lin & Li, 2021 (♂)	China
*Meotipamultuma* Murthappa, Malamel, Prajapati, Sebastian & Venkateshwarlu, 2017 (♂♀)	India
*Meotipapallida* Deeleman-Reinhold, 2009 (♀)	Indonesia
*Meotipapicturata* Simon, 1895 (♂♀)	India, Thailand, Laos, Indonesia
*Meotipapulcherrima* (Mello-Leitão, 1917) (♂♀)	tropical Africa, introduced to the Americas, Papua New Guinea, China, Korea, Japan, and Pacific Islands
*Meotipasahyadri* Kulkarni, Vartak, Deshpande & Halali, 2017 (♂♀)	India
*Meotipaspiniventris* (O. Pickard-Cambridge, 1869) (♂♀)	Sri Lanka to Taiwan, China, Japan, introduced to the Netherlands
*Meotipathalerorum* Deeleman-Reinhold, 2009 (♂♀)	Malaysia and Indonesia
*Meotipaultapani* Basumatary & Brahma, 2019 (♀)	India
*Meotipavesiculosa* Simon, 1895 (♂♀)	China, Vietnam to Japan, Philippines, Indonesia
*Meotipazhengguoi* Lin & Li, 2021 (♂)	China

#### ﻿ Meotipa argyrodiformis

Taxon classificationAnimaliaAraneaeTheridiidae

(Yaginuma, 1952)

8FFA37F6-D4CD-5DD7-A969-1424403A5196

Figures 1A–H , 10

Ariamnes argyrodiformis Yaginuma, 1952: 14, figs 1–6 (description of female).
Topo argyrodiformis Yaginuma, 1955: 16 (transferred from Ariamnes, at the time in synonymy with Argyrodes).
Chrysso argyrodiformis Yaginuma, 1965: 35 (transferred from Topo, at the time in synonymy with Thwaitesia); Yaginuma 1986: 45, fig. 24.3 (female, description of male); Chikuni 1989: 32, fig. 14 (male and female); Chen and Gao 1990: 93, fig. 116 (female); Barrion and Litsinger 1995: 419, figs 248a–i, 249a, b (male and female); Yoshida 2003: 128, figs 338, 343–345, 584 (male and female); Yin et al. 2012: 293, figs 106a–d (female); Sen et al. 2015: 85, figs 493–498, pl. 19 (female).
Meotipa argyrodiformis Yoshida, 2009: 378, figs 208–210 (transferred from Chrysso).

##### Material examined (holotype not examined).

**Hunan Province**: 9♀, Changsha City, Yuelu Mountain Scenic Area (28.19°N, 112.94°E, 210 m), 12 August 2018, Z.C. Li & Z.W. Deng leg. (CBEE).

##### Diagnosis.

**Figure 1.**
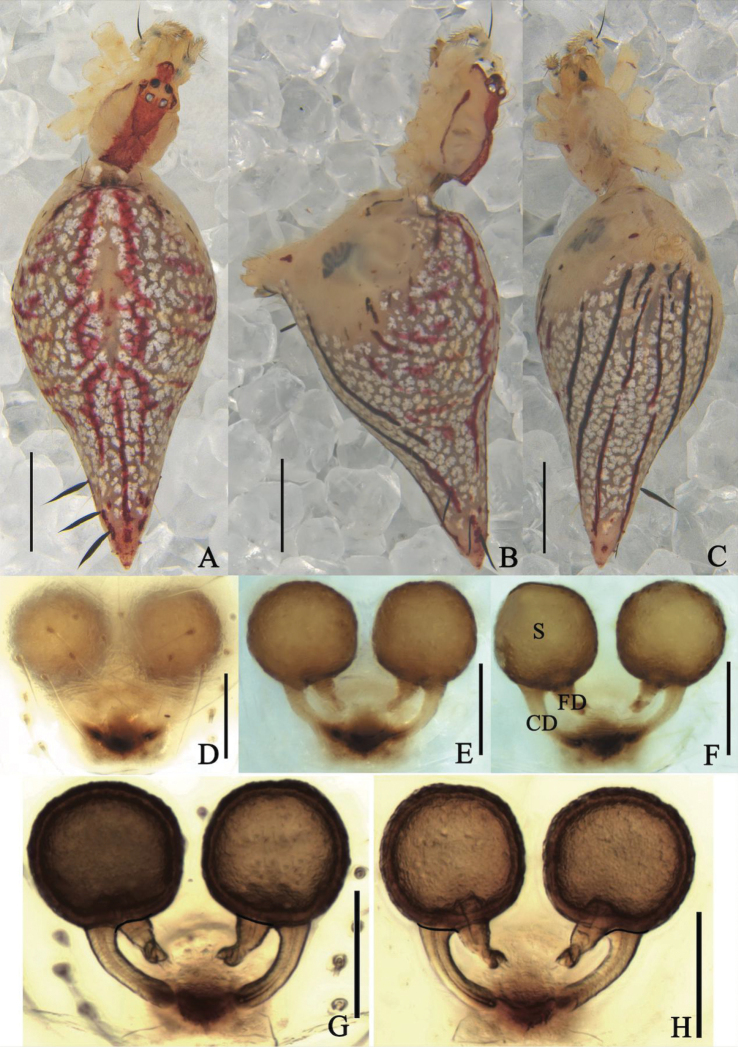
*Meotipaargyrodiformis* (Yaginuma, 1952) **A–C** female habitus (some flattened black spines on the abdomen and legs were broken off during photography) (**A** dorsal **B** prolateral **C** ventral). **D–H** epigynum (**D** ventral, on the body **E, F** in alcohol **E** ventral **F** dorsal **G, H** in gum arabic **G** ventral **H** dorsal). Scale bars: 1 mm **(A–C)**; 0.1 mm **(D–H)**. Abbreviations: CD = copulatory duct, FD = fertilization duct, S = spermathecae.

**Figure 2.**
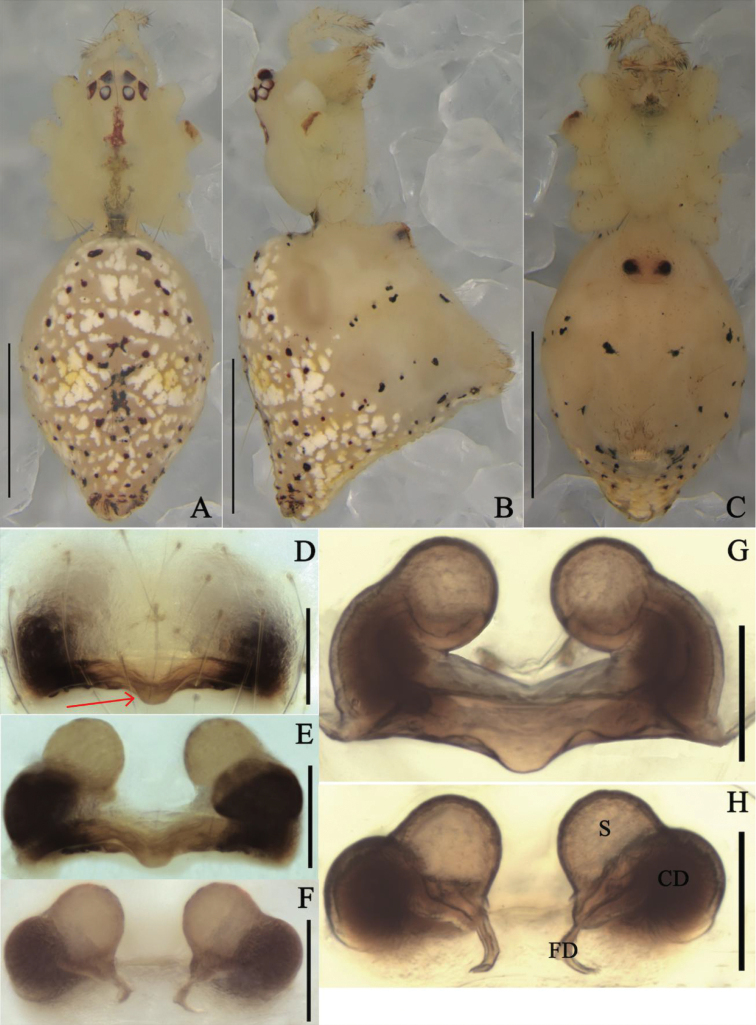
*Meotipapulcherrima* (Mello-Leitão, 1917) **A–C** female habitus (flattened black spines on the abdomen and legs were broken off during photography) (**A** dorsal **B** prolateral **C** ventral). **D–H** epigynum (**D** ventral, on the body, and the arrow points to tongue-shaped apophysis **E, F** in alcohol **E** ventral **F** dorsal **G, H** in gum arabic **G** ventral **H** dorsal). Scale bars: 1 mm **(A–C)**; 0.1 mm **(D–H)**. Abbreviations: CD = copulatory duct, FD = fertilization duct, S = spermathecae.

**Figure 3.**
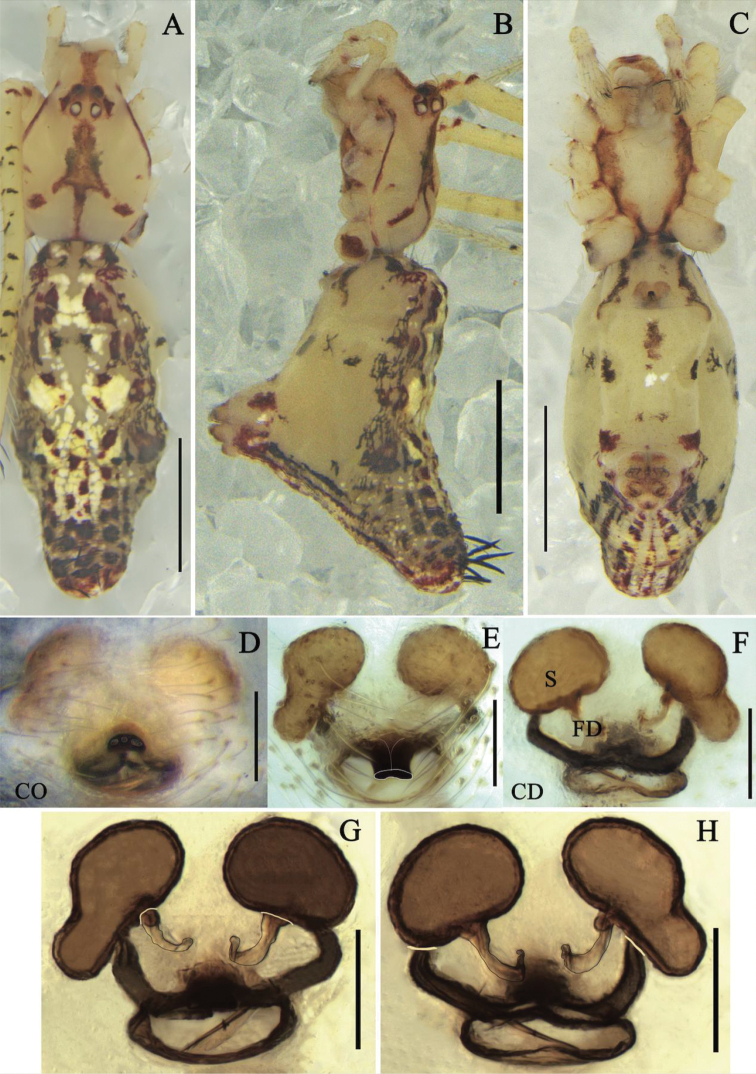
*Meotipapicturata* Simon, 1895 **A–C** female habitus **(A** dorsal **B** prolateral **C** ventral). **D–H** epigynum (**D** ventral, on the body **E, F** in the alcohol **E** ventral **F** dorsal **G, H** in gum arabic **G** ventral **H** dorsal). Scale bars: 1 mm **(A–C)**; 0.1 mm **(D–H)**. Abbreviations: CD = copulatory duct, FD = fertilization duct, S = spermathecae.

**Figure 4.**
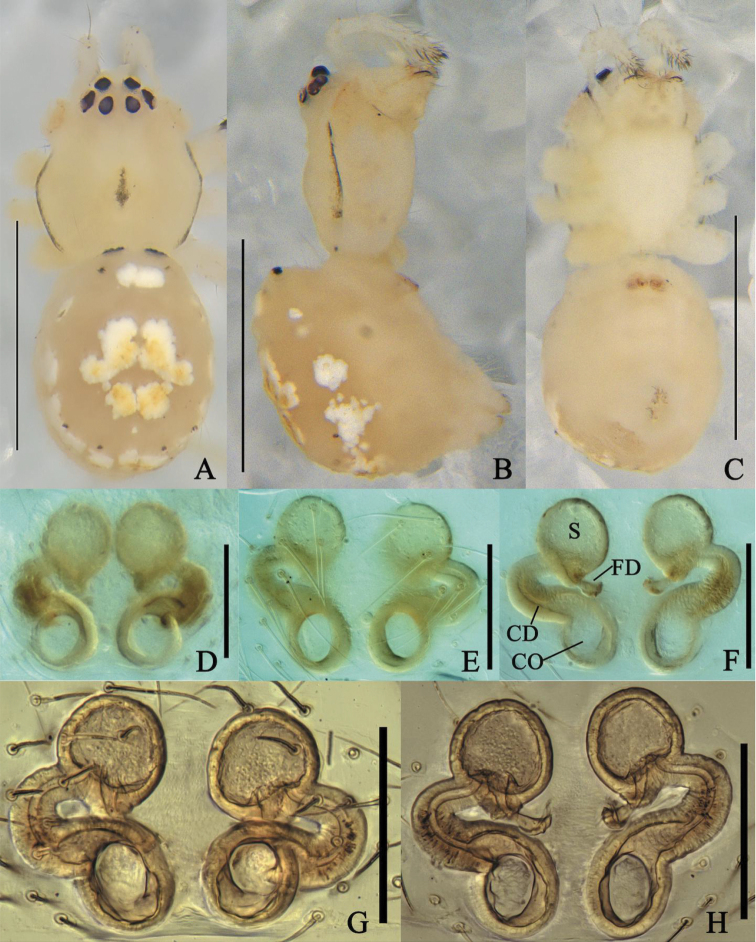
*Meotipaspiniventris* (O. Pickard-Cambridge, 1869) **A–C** female habitus (flattened black spines on the abdomen and legs were broken off during taking photographs) (**A** dorsal **B** Prolateral **C** ventral). **D–H** epigynum (**D** ventral, on the body **E, F** in the alcohol **E** ventral **F** dorsal **G, H** in gum arabic **G** ventral **H** dorsal). Scale bars: 1 mm **(A–C)**; 0.1 mm **(D–H)**. Abbreviations: CO = copulatory opening, CD = copulatory duct, FD = fertilization duct, S = spermathecae.

*Meotipaargyrodiformis* is similar to *M.pulcherrima* (Fig. 2A–H) in: having the abdomen pointed to a tubercle posteriorly (Fig. 1B), lacking obvious copulatory openings, and having a spoon-shaped conductor in the male palp. However, it can be distinguished from the latter by the following combination of characters: (1) the abdomen extends beyond spinnerets ~ 3/4 of total abdomen length in *M.argyrodiformis* (Fig. 1A), but less than half in *M.pulcherrima* (Fig. 2A); (2) the epigynum does not have a tongue-shaped apophysis posteriorly and medially in *M.argyrodiformis* (Fig. 1D), but has in *M.pulcherrima* (Fig. 2D); (3) the copulatory ducts are tube-shaped in *M.argyrodiformis* (Fig. 1F), but spherical in *M.pulcherrima* (Fig. 2F); (4) the cymbium is long and nearly ovoid in *M.argyrodiformis* (see Yoshida 2003: fig. 345), but shorter and nearly spherical in *M.pulcherrima* (see Yoshida 2003: fig. 342); (5) the embolus is long with a twisted tip, extending beyond conductor in *M.argyrodiformis* (see Yoshida 2003: fig. 345), but short and straight, completely surrounded by conductor in *M.pulcherrima* (see Yoshida 2003: fig. 342).

##### Description.

**Female.** Total length 5.20; Prosoma length 1.20, width (at middle) 0.81, height (at middle) 0.88; Opisthosoma length 4.05, width (at middle) 1.97, height (at middle) 2.46; Eye diameters: ALE 0.07, AME 0.07, PLE 0.07, PME 0.07; Eye interdistances: AME–AME 0.05, ALE–ALE 0.15, PLE–ALE contiguous, PLE–PLE 0.22, PME–PME 0.07, PME–PLE 0.07, AME–ALE 0.05; Clypeus height (at middle) 0.38, width (at middle) 0.16; Measurements of legs: Leg I (right) 11.18 [4.93, 0.84, 2.36, 2.34, 0.71], II (right) 6.72 [1.85, 0.49, 1.50, 2.17, 0.71], III (right) 4.61 [1.44, 0.38, 0.85, 1.42, 0.52], IV (right) 9.27 [2.80, 0.59, 2.69, 2.43, 0.76]. Carapace rather ovate with V-shaped longitudinal fovea, cephalic area short and narrow, thoracic area widest between coxae II and III (Fig. 1A). Eyes in two rows with black or pale brown ring, strongly recurved (Fig. 1B); anterior eye row curved, shorter than the straight posterior eye row. Sternum yellow, longer than wide, lateral margins slightly indented. Labium contiguous to the sternum, yellow with a brown, rounded to slightly triangular, apical margin (Fig. 1C). Chelicerae vertical, yellow with pale brown fang. Yellow legs long and slender with or without gray bands and bearing a few dark brown short spines, tibiae with two dorsal spines in legs I, II and IV, and one in leg III, tarsus three-clawed with 5–7 teeth in the superior claws. Leg formula 1423. Pedipalp yellow and length ~ 2/3 carapace, single-clawed, with many short hairs; tibia laterally with a lanceolate spine. Abdomen pale yellowish, anterior portion overhangs the carapace, approximately triangular in lateral view, dorsum with red cardiac marking and several transverse reddish brown bands laterally. Posterior area of ventral abdomen with deep red stripes, pointed posteriorly, with six black and medially broad setae with sharp ends, and ventrally projected spinnerets (Fig. 1B). Epigynum forming a triangular atrium, without obvious copulatory openings (Fig. 1D); two round spermathecae can be clearly seen in ventral view (Fig. 1E); copulatory ducts long, narrow, almost straight; fertilization ducts short, close to copulatory ducts, and both of them are located at the directly below spermathecae (Fig. 1F); spermathecae round, slightly separate from each other (Fig. 1H).

**Male.** Not collected.

##### Distribution.

China (Anhui, Fujian, Hunan, Liaoning, Shaanxi, Sichuan, Taiwan, Zhejiang), Japan, Philippines, India.

#### ﻿ Meotipa pulcherrima

Taxon classificationAnimaliaAraneaeTheridiidae

(Mello-Leitão, 1917)

E7C7E9D8-1F14-59D4-908F-45A42504858E

Figures 2A–H , 9 , 10

Argyrodes pulcherrimus Mello-Leitão, 1917: 86, figs 7, 8 (description of female).
Meotipa clementinae Petrunkevitch, 1930: 212, figs 61, 62 (description of female); Schmidt 1956a: 30, fig. 6 (description of male); Schmidt, 1956b: 240, fig. 1 (female).
Argyrodes elevatus Exline and Levi, 1962: 135 (synonymy, rejected by Levi 1967a).
Chrysso clementinae Levi, 1962: 231, figs 71–75 (male and female); Müller 1992: 99, figs 5, 6 (female).
Chrysso pulcherrima Levi, 1967a: 26 (removed female from synonymy of Argyrodeselevatus, synonymy of male); Levi 1967b: 182, figs 28–31 (male and female); Zhu and Zhang 1992: 23, fig. 3A–D (male and female); Yoshida 1993: 30, figs 10–12, 20 (male and female); Zhu 1998: 54, fig. 28A–D (male and female); Song et al. 1999: 103, fig. 50A, B, J (male and female); Yoshida 2003: 126, figs 337, 341, 342 (male and female, synonymy); Seo 2005: 123, fig. 2A, B (male).
Chrysso mussau Chrysanthus, 1975: 48, figs 174–177 (descriptions of male and female).
Meotipa pulcherrima Yoshida, 2009: 378, figs 211–213 (transferred from Chrysso).

##### Note.

The taxonomy of *M.pulcherrima*, presumed to be widely introduced, including to its type locality in Brazil, requires further scrutiny. Our specimens are not identical to the given type illustrations, and further variation appears globally evident. However, solving the global taxonomy of *M.pulcherrima* is outside the scope of this manuscript and required dedicated research.

##### Material examined (holotype not examined).

**Hunan Province**: 7♀, Zhangjiajie City, Sangzhi County, Quanyushan Leisure Park (29.48°N, 110.16°E, 370 m), 5 May 2018, F.X. Liu & Z.C. Li leg. (CBEE).

##### Diagnosis.

*Meotipapulcherrima* is similar to *M.capacifaba* Li, Liu, Xu & Yin, 2020 (see Li et al. 2020: figs 1A–J, 2A–E, 3A–E) by shared characters such as raised eyes, raised carapace posteriorly, and spherical copulatory ducts (Fig. 2G), but it can be distinguished from the latter by the following characters: (1) the copulatory openings are inconspicuous in *M.pulcherrima* (Fig. 2D), but obvious in *M.capacifaba* (see Li et al. 2020: fig. 2A); (2) the posterior margin of atrium has a tongue-shaped protrusion medially in *M.pulcherrima* (Fig. 2E), but not in *M.capacifaba* (see Li et al. 2020: fig. 2A); (3) the conductor is broad with a sharp point in *M.pulcherrima* (see Levi 1962: figs 74, 75), but relatively narrow with a blunt point in *M.capacifaba* (see Li et al. 2020: figs 2D, 3D).

##### Description.

**Female.** Total length 3.03; Prosoma length 0.96, width (at middle) 0.90, height (at middle) 0.61; Opisthosoma length 2.07, width (at middle) 1.27, height (at middle) 1.89; Eye diameters: ALE 0.07, AME 0.09, PLE 0.08, PME 0.09; Eye interdistances: AME–AME 0.09, ALE–ALE 0.55, PLE–ALE contiguous, PLE–PLE 0.30, PME–PME 0.08, PME–PLE 0.11, AME–ALE 0.02; Clypeus height (at middle) 0.21, width (at middle) 0.60; Measurements of legs: Leg I (right) 8.3 [2.59, 0.42, 2.34, 2.16, 0.79], II (right) 5.42 [2.03, 0.40, 1.04, 1.43, 0.52], III (right) 3.73 [1.49, 0.24, 0.85, 0.73, 0.42], IV (right) 6.33 [2.40, 0.39, 0.90, 2.00, 0.64]. Carapace with a central reddish brown longitudinal band; cephalic area relatively long and narrow; clypeus bulged outward (Fig. 2A). Median furrow is round, deep, and the radial furrow is not obvious (Fig. 2A). Eyes strongly recurved. AME separation is greater than AME-ALE, and PME separation is also greater than PLE-PME; ALE-PLE contiguous. All eyes nearly uniform in size with brown rings surrounding (Fig. 2A, B). Sternum yellow, heart-shaped. Labium clearly distinguish from the sternum, yellow with brown markings, approximately triangular in shape. Chelicera yellow with pale red fang (Fig. 2C). Yellow legs long and slender with dark brown spots; the ends of tibiae and the bases and ends of metatarsus of each leg have dark brown rings. Leg formula 1423. Pedipalp yellow with many short hairs distally (Fig. 2A, B). Abdomen triangular in lateral view, with caudal region extending upwardly beyond spinnerets. There are 14 feather-like spines on the top of the protuberance and before the protuberance reaches the spinnerets, which break easily (Fig. 2A–C). Dorsum of abdomen yellow with dark red spots. The venter of the abdomen has central black spots on each side (Fig. 2B). Epigynum with a big atrium and inconspicuous copulatory openings (Fig. 2D); copulatory ducts swelling distally, sphere-shaped (Fig. 2E, F); spermathecae smaller than distal end of copulatory ducts, sphere-shaped (Fig. 2H); fertilization ducts are located at the intersection of copulatory ducts and spermathecae (Fig. 2H).

**Male.** Not collected.

##### Distribution.

China (Fujian; Guangxi; Hainan; Hunan, newly recorded; Taiwan; Zhejiang), Japan, Korea, Pacific Is., Papua New Guinea; also tropical Africa and widespread across the Americas (after Levi 1962).

##### Remarks.

Although we did not examine the female holotype of *M.pulcherrima*, the triangular abdomen with caudal region extending upwardly beyond spinnerets, the short and swollen copulatory ducts, and the sphere-shaped spermathecae all indicate our specimens belong to *M.pulcherrima* according to the original, albeit simple illustrations by Mello-Leitão (1917: 86, figs 7, 8) and detailed illustrations by Zhu et al. (1992: 23, fig. 3A–D) and Yoshida (2003: 126, figs 337, 341, 342).We also note some slight differences in our specimens against the original illustrations of Brazilian specimens by Mello-Leitão (1917). The specimens we collected only has half of the abdomen extending beyond the spinnerets (Fig. 2B), but the specimens from Brazil have 2/3 of abdomen extending beyond them (see Mello-Leitão 1917: 86, fig. 7). Meanwhile, the shadow of the copulatory ducts and the fertilization ducts can be seen from the ventral view of epigynum, and the shadows on both sides look like two tadpoles in atrium, with fertilization ducts resembling their tails and copulatory ducts resembling their heads. The shadows of the specimen from Brazil in 1917 resemble two tadpoles swimming towards the middle of the atrium (see Mello-Leitão 1917: 86, fig. 8), but the shadows of our specimen resemble two tadpoles swimming to the sides of the atrium (Fig. 2C). However, the tails of tadpoles in the Brazilian specimens may be the spermathecae instead of the fertilization ducts according to the original illustrations. In addition, the posterior margin of atrium has a tongue-shaped protrusion medially in our individuals, but not in the Brazilian specimens.

#### ﻿ Meotipa picturata

Taxon classificationAnimaliaAraneaeTheridiidae

Simon, 1895

7E076A61-D6DD-54A6-88FE-4BF4BA6FD94D

Figures 3A–H , 9 , 10

Meotipa picturata Simon, 1894: 519 (nomen nudum); Simon, 1895: 133 (description of female); Levi and Levi 1962: 47, figs 112–113 (female); Deeleman-Reinhold, 2009: 410, figs 1–3 (female); Kulkarni et al. 2017: 515, figs 39–44 (female); Murthappa et al. 2017: 590, figs 1A–J, 2A–F, 4A–D (description of male, female).

##### Material examined (holotype not examined).

**Yunnan Province**: 1♀, Mengla County, Menlun Town, Xishuangbanna Tropical Botanical Garden, Chinese Academy of Sciences (21.92°N, 101.26°E, 550 m), 22 November 2019, J. Chen, J. Liu, Z.C. Li & B. Liang leg. (CBEE).

##### Diagnosis.

*Meotipapicturata* is similar to *M.sahyadri* (Kulkarni et al. 2017: figs 1–38) in having a deep round atrium, the presence of rod-shaped projection with distinct oval openings distally in the median of the atrium (Fig. 3D). It can be distinguished from the latter by the following combination of characters: (1) the rod-shaped projection with three openings in this species (Fig. 3D), but two openings in *M.sahyadri* (see Kulkarni et al. 2017: figs 19, 22); (2) the distal shape of rod-shaped projection is flat, quadrangular in *M.picturata* (Fig. 3E), but triangular in *M.sahyadri* (see Kulkarni et al. 2017: figs 19, 21); (3) the copulatory ducts curve outward in *M.picturata* (Fig. 3F), but curve inward in *M.sahyadri* (see Kulkarni et al. 2017: fig. 18); (4) the end of copulatory ducts enter into the spermathecae ventrally in *M.picturata* (Fig. 3E), but laterally in *M.sahyadri* (see Kulkarni et al. 2017: fig. 18).

##### Description.

**Female.** Total length 3.78; Prosoma length 1.31, width (at middle) 0.94, height (at middle) 0.47; Opisthosoma length 2.48, width (at middle) 1.17, height (at middle) 1.94; Eye diameters: ALE 0.08, AME 0.08, PLE 0.08, PME 0.08; Eye interdistances: AME–AME 0.06, ALE–ALE 0.15, PLE–ALE contiguous, PLE–PLE 0.19, PME–PME 0.09, PME–PLE 0.05, AME–ALE 0.04; Clypeus height (at middle) 0.31, width (at middle) 0.20; Measurements of legs: Leg I (right) 13.96 [4.74, 0.49, 3.02, 4.71, 1.00], II (right) 8.88 [3.02, 0.51, 1.69, 2.86, 0.80], III (right) 7.87 [2.28, 0.26, 0.94, 1.53, 2.86], IV (right) 12.19 [4.14, 0.32, 2.96, 3.96, 0.81]. Prosoma glabrous; clypeus narrow. Carapace with a central arrowed-shaped marking forward; fovea broad, smooth with distinct depression (Fig. 3A). All eyes nearly uniform in size. Sternum triangular, yellow medially, lateral margins deep red and indented. Labium contiguous to the sternum, short with small hairs. Chelicera slanting, yellow with red fang (Fig. 3A–C). Yellow legs long and slender with short hairs. Tibia with lanceolate spines and black ring distally; femur with black spots dorsally and red spots distally. Leg formula 1423. Pedipalp yellow with small hairs; tibia distally with red semicircle ventrally; tarsus with a single claw. Opisthosoma triangular laterally, very broad in lateral view, its dorsum provided with alternate red and yellow spots, posterior region knobbed extending upwardly, provided with lanceolate spines. Venter transparent, some small red, yellow, and white patches posteriorly (Fig. 3B). Atrium broad, with a rod-shaped projection apically (Fig. 3D); copulatory openings depressed, contiguous, located on the end of rod-shaped projection (Fig. 3E); copulatory ducts broad, short, tube extensively curved entering into the base of spermathecae (Fig. 3F); spermathecae asymmetrical in shape, left spermatheca gourd-shaped, right spermatheca oval; fertilization duct long, originating from the medial of basal spermathecae (Fig. 3H).

**Male.** Not collected.

##### Distribution.

China (Yunnan). New country and province record (Fig. 10).

#### ﻿ Meotipa spiniventris

Taxon classificationAnimaliaAraneaeTheridiidae

(O. Pickard-Cambridge, 1869)

8A912E94-845B-57DA-AC33-5171A53CEAE8

**Figure 5.**
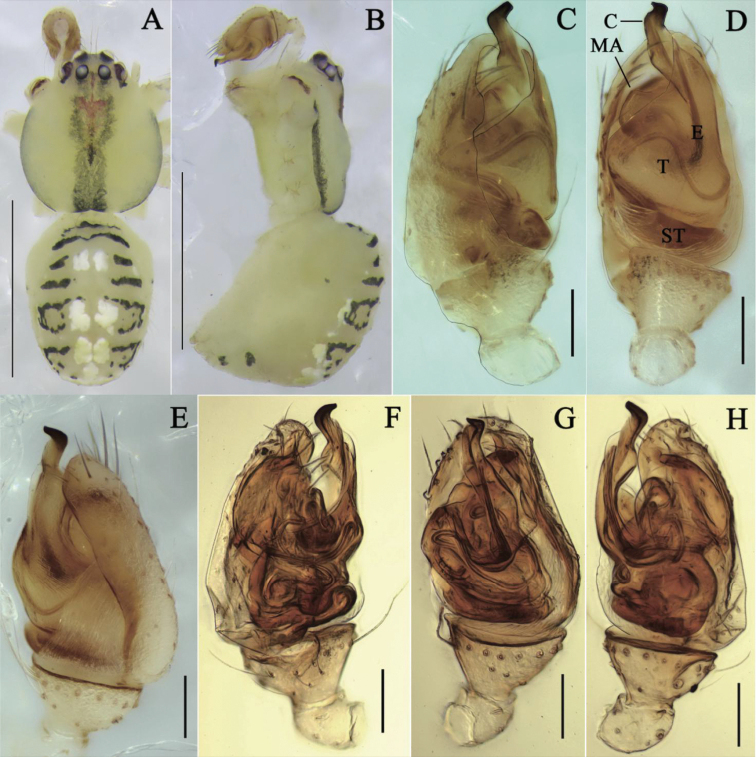
*Meotipaspiniventris* (O. Pickard-Cambridge, 1869) **A–B** male habitus (flattened black spines on the abdomen were absolutly absent) (**A** dorsal **B** prolateral). **C–H** pedipalp (**C–E** in the alcohol **C** prolateral **D** ventral **E** retrolateral **F–H** in gum arabic **F** prolateral **G** ventral **H** retrolateral). Scale bars: 1 mm **(A–C)**; 0.1 mm **(D–I)**. Abbreviations: E = embolus, MA = median apophysis, ST = subtegulum, T = tegulum.

Figures 4A–H , 5A–H , 9 , 10

Theridion spiniventre O. Pickard-Cambridge, 1869: 384, pl. 12, figs 52–56 (description of male); Hammen 1949: 76, figs 1–3 (male and female); Yoshida 1977: 9, figs 1–4 (male and female); Song 1987: 128, fig. 89 (male and female).
Theridion buitenzorgi Strand, 1907: 412 (female).
Chrysso spiniventre Yaginuma, 1986: 46, fig. 24–5 (transferred from Theridion); Zhu 1998: 66, fig. 38A–D (male and female); Song et al. 1999: 107, fig. 50E–L (male and female); Yoshida 2003: 130, figs 346–350 (male and female).
Meotipa spiniventris Yoshida, 2009: 378, figs 214–216 (transferred from Chrysso).

##### Material examined (holotype not examined).

**Yunnan Province**: 1♀, Mengla County, Menlun Town, Xishuangbanna Botanical Garden, Chinese Academy of Sciences (21.93°N, 101.25°E, 570 m), 22 March 2018, F.X. Liu & Z.C. Li leg. (CBEE); **Hainan Province**: 1♂, 5♀, Wuzhishan City, Shuiman Township, Wuzhishan (18.88°N, 109.66°E, 140 m), 15 April 2018, F.X. Liu & Z.C. Li leg. (CBEE); 3 ♀, Wuzhishan City (18.78°N, 109.52°E, 350 m), 15 April 2018, F.X. Liu & Z.C. Li leg. (CBEE); 2 ♀, Wuzhishan City, Diaoluo Mountain (18.78°N, 109.52°E, 136 m), 17 April 2018, F.X. Liu & Z.C. Li leg. (CBEE); **Sichuan Province**: 1♀, Ya’an City, Lushan County, Longmen Town (30.25°N, 103.02°E, 810 m), 28 September 2018, F.X. Liu, Z.C. Li & Z.W. Deng leg. (CBEE); 1♀, Chengdu City, Qinglong Lake Park (30.65°N, 104.20°E, 490 m), 30 September 2018, F.X. Liu, Z.C. Li & Z.W. Deng leg. (CBEE); 1♀, Chengdu City, Tazishan Park (30.64°N, 104.12°E, 490 m), 30 September 2018, F.X. Liu, Z.C. Li & Z.C. Deng leg. (CBEE); **Hubei Province**: 1♀, Jianshi Country, Chaoyang Temple (30.60°N, 109.71°E, 490 m), 7 October 2018, F.X. Liu, Z.C. Li & Z.W. Deng leg. (CBEE).

##### Diagnosis.

*Meotipaspiniventris* is similar to *M.multuma*Murthappa et al., 2017 (see Murthappa et al. 2017: figs 3A–E, 4E, F) in shared characters such as raised eyes, forward slanting clypeus and two separated copulatory openings. Females of *M.spiniventris* can be distinguished from *M.multuma* by the following combination of characters: (1) the carapace has a central black band, opisthosoma with four median yellow and white patches, and four or five lanceolate spines posteriorly in *M.spiniventris* (Fig. 4A), but carapace without any marking, opisthosoma with a median black patch and 12–15 lanceolate spines posteriorly in *M.multuma* (see Murthappa et al. 2017: fig. 3A–C); (2) copulatory ducts short, extending to the lateral part of spermathecae (Fig. 4E) and fertilization ducts short in *M.spiniventris* (Fig. 4F, H), but the copulatory ducts are long and convoluted around spermathecae and fertilization ducts longer, twisted in *M.multuma* (see Murthappa et al. 2017: figs 3D, E, 4E, F); (3) the tip of the conductor is uniquely strongly sclerotized and twisted in *M.spiniventris* (Fig. 5C).

##### Description.

**Female.** Total length 2.23; Prosoma length 1.03, width (at middle) 0.71, height (at middle) 0.51; Opisthosoma length 1.20, width (at middle) 0.81, height (at middle) 1.05; Eye diameters: ALE 0.06, AME 0.07, PLE 0.06, PME 0.07; Eye interdistances: AME–AME 0.06, ALE–ALE 0.11, PLE–ALE contiguous, PLE–PLE 0.17, PME–PME 0.07, PME–PLE 0.05, AME–ALE 0.02; Clypeus height (at middle) 0.15, width (at middle) 0.13; Measurements of legs: Leg I (right) 9.19 [2.95, 0.47, 2.09, 2.93, 0.75], II (right) 4.83 [1.77, 0.36, 1.09, 1.16, 0.45], III (right) 2.98 [1.20, 0.22, 0.50, 0.72, 0.34], IV (right) 6.57 [2.21, 0.37, 1.21, 1.29, 1.49]. Carapace rhomboid with narrow and bar-shaped longitudinal fovea, glabrous; cephalic area relatively long and broad; clypeus slightly elevated. Eye field raised, all eyes in two rows and nearly uniform in size (Fig. 4A). Sternum white, subtly heart-shaped. Labium contiguous with the sternum, white with brown, approximately triangular in shape. Chelicera slanting, white with black fang. Yellow legs long and slender with several black lanceolate spines and the top of tarsus, metatarsus and tibia have black ring in leg I. Leg formula 1423. Yellow pedipalp with short hairs (Fig. 4A–C). Opisthosoma oval higher than long. Abdomen with four black and medially broad setae, dorsum provided with yellow and white (Figs 4B, C). Venter transparent, without any marking (Fig. 4C). Two copulatory openings not juxtaposed (Fig. 4D); copulatory ducts short, inward curving almost 180° and extending to lateral sides of spermathecae (Fig. 4F); spermathecae oval-shaped; fertilization ducts located on the base of spermathecae (Fig. 4H).

**Male.** Total length 1.82; Prosoma length 0.80, width (at middle) 0.70, height (at middle) 0.54; Opisthosoma length 1.02, width (at middle) 0.93, height (at middle) 0.83; Eye diameters: ALE 0.06, AME 0.08, PLE 0.07, PME 0.07; Eye interdistances: AME–AME 0.08, ALE–ALE 0.15, PLE–ALE contiguous, PLE–PLE 0.19, PME–PME 0.07, PME–PLE 0.06, AME–ALE 0.04; Clypeus height (at middle) 0.19, width (at middle) 0.15; Measurements of legs: Leg I (right) 11.08 [3.36, 0.44, 2.83, 3.52, 0.93], II (right) 6.1 [1.62, 0.36, 1.54, 1.87, 0.71], III (right) 3.55 [1.24, 0.21, 0.74, 0.97, 0.39], IV (right) 7.87 [2.92, 0.38, 2.08, 1.88, 0.61]. Like the female, except by the following. Dwarf in size compared to female (4/5 size of female), without characteristic lanceolate spines. Prosoma with distinct black streaks medially, and a wide red streak in the front of the black streaks; clypeus slightly bulged; eye field wide, elevated, with two long hairs in the middle of AME, white appearance except bulged out black anterior medians (Fig. 5A). Sternum heart shaped; maxillae, labium with dense tuft of hairs; Leg formula 1423 (Fig. 5B). Opisthosoma with alternate deep green and white patches, slightly high, without humps (Fig. 5A). Tegulum spherical, with median apophyses (Fig. 5D); conductor transparent, spirals upward beyond cymbium, with distal end strongly sclerotized (Fig. 5F); embolus long, narrow, with a sharp tip, almost completely covered by conductor (Fig. 5H).

##### Distribution.

China (Jiangxi; Hainan, newly recorded; Hubei, newly recorded; Sichuan, newly recorded; Yunnan, newly recorded; Taiwan), Japan, Netherlands, Sri Lanka.

#### ﻿ Meotipa vesiculosa

Taxon classificationAnimaliaAraneaeTheridiidae

Simon, 1895

7865D7EE-4516-56E2-95DA-9123B8D461AC

Figures 6A–H , 10

Meotipa vesiculosa Simon, 1894: 514, figs 522, 527 (nomen nudum); Simon 1895: 134 (description of female); Deeleman-Reinhold 2009: 415, figs 13–19 (transferred from Chrysso); Yoshida 2009: 378, figs 205–207 (male and female).
Chrysso vesiculosa Levi, 1962: 232, figs 80, 81 (female); Yaginuma 1986: 45, fig. 24–1 (female); Chikuni 1989: 32, fig. 15 (female); Zhu 1998: 51, fig. 25A–C (female); Song et al. 1999: 107, fig. 51E, F (female); Yoshida 2003: 125, figs 336, 339–340, 583 (female); Yoshida 2006: 23, figs 1–7 (female); Yin et al. 2012: 305, fig. 115a–f (male and female).
Chrysso jianglensis Zhu & Song, in Song et al. 1993: 857, fig. 9A–C (description of male); Zhu 1998: 68, fig. 39A–C (male); Song et al. 1999: 103, fig. 49K, L (male).

##### Material examined (holotype not examined).

**Yunnan Province**: 4♀, Mengla County, Menlun Town, Xishuangbanna Botanical Garden, Chinese Academy of Sciences (21.93°N, 101.25°E, 570 m), 22 March 2018, F.X. Liu & Z.C. Li leg. (CBEE); **Hunan Province**: 4♀, Changsha City, Yuelu Mountain Scenic Area (28.19°N, 112.94°E, 210 m), 12 August 2018, Z.C. Li & Z.W. Deng leg. (CBEE); 1♀, Hengyang City, Hengshan Mountain Scenic Area (27.27°N, 112.71°E, 1300 m), 17 August 2018, Z.C. Li & Z.W. Deng leg. (CBEE).

##### Diagnosis.

**Figure 6.**
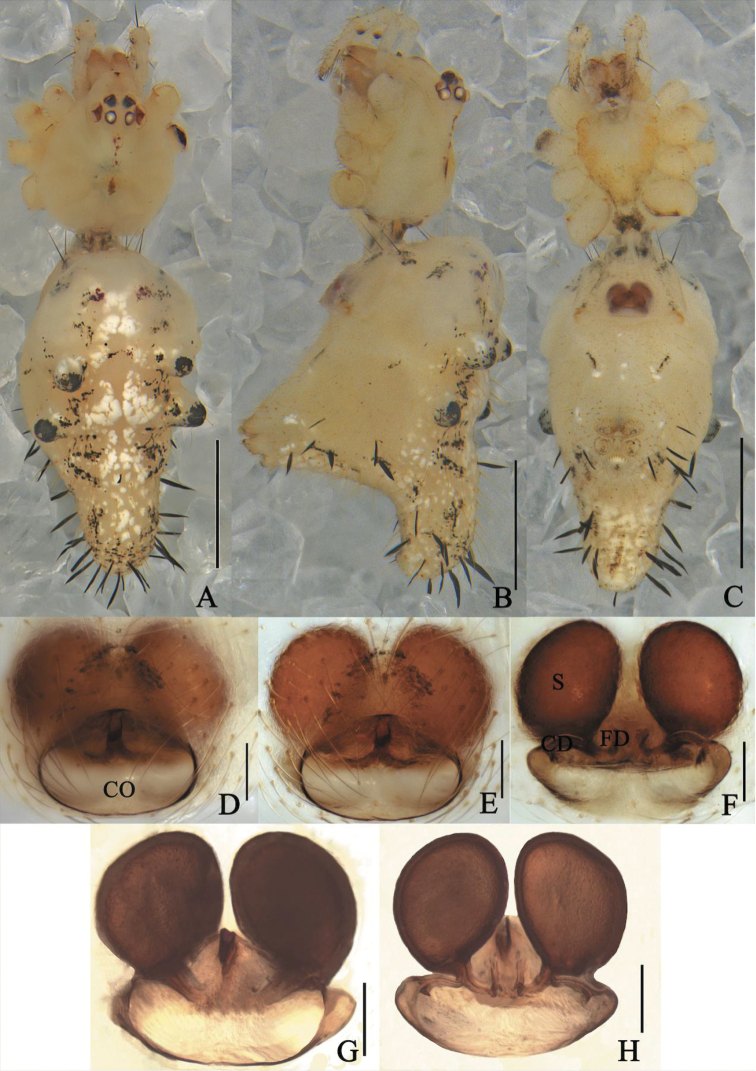
*Meotipavesiculosa* Simon, 1895 **A–C** female habitus (**A** dorsal **B** prolateral **C** ventral). **D–H** epigynum (**D** ventral, on the body **E, F** in the alcohol **E** ventral **F** dorsal **G, H** in gum arabic **G** ventral **H** dorsal). Scale bars: 1 mm **(A–C)**; 0.1 mm **(D–H)**. Abbreviations: CO = copulatory opening, CD = copulatory duct, FD = fertilization duct, S = spermathecae.

This species can be distinguished from other *Meotipa* species by the combination of following characters: (1) atrium large, with shallow heart-shaped depression (Fig. 6D); (2) lateral edges of spermathecae aligned with copulatory ducts (Fig. 6H); (3) conductor expanding laterally converting to ring-shape distally (see Yoshida 2009: fig. 207); (4) embolus incurved and long, knife shaped distally (see Yin et al. 2012: fig. 115e, f).

##### Description.

**Female.** Cephalothorax pale yellow with a red-brown central stripe; cephalic area relatively long and broad; clypeus narrow, bulged out. Eyes in two rows and nearly uniform in size, strongly recurved; AME separation is greater than AME-ALE, and PE are arranged at almost equal distances; AME black, PME eyes pearly white (Fig. 6A). Sternum deep yellow, triangular, lateral margins slightly indented. Labium contiguous with the sternum, brown, triangular. Chelicera vertical, deep yellow with red fang (Fig. 6B). Legs yellowish white with small hairs and bearing a few dark short spines, the end of tibiae with a black ring in each legs and femur of all legs have small black spots. Leg formula 1423. Pedipalp yellowish white, single-clawed, with many short hairs; tibia with a black ring on the extreme and bearing a lanceolate spine laterally (Fig. 6A, B). Opisthosoma approximately triangular laterally, dorsally provided with numerous white and black spots, posterior region extending downwardly towards spinnerets. Two pairs of dorsolateral abdominal humps, black distally (Fig. 6A–C). Atrium large, with a clear herringbone structure medially (Fig. 6D). Copulatory ducts and fertilization ducts short and both of them extending into spermathecae at the same position (Fig. 6H). Spermathecae large, oval-shaped (Fig. 6G).

**Male.** Not collected.

##### Distribution.

China (Fujian; Guangxi; Hunan; Taiwan; Yunnan, newly recorded), Vietnam to Japan, Philippines, Indonesia.

#### ﻿ Meotipa pseudopicturata sp. nov.

Taxon classificationAnimaliaAraneaeTheridiidae

6514A605-D9D2-5D1C-B5A5-F709476AAFAD

http://zoobank.org/E6771BF5-0A42-44A0-B57D-19008459EB38

Figures 7A–H , 10

##### Type material.

***Holotype*** female (CBEE). **Yunnan Province**: Mengla County, Menlun Town, Xishuangbanna Botanical Garden, Chinese Academy of Sciences (21.93°N, 101.25°E, 570 m), 22 March 2018, F.X. Liu & Z.C. Li leg. ***Paratypes***. **Yunnan Province**: 7♀, same data as holotype.

##### Etymology.

The species epithet refers to its similarity to *Meotipapicturata* Simon, 1895.

##### Diagnosis.

**Figure 7.**
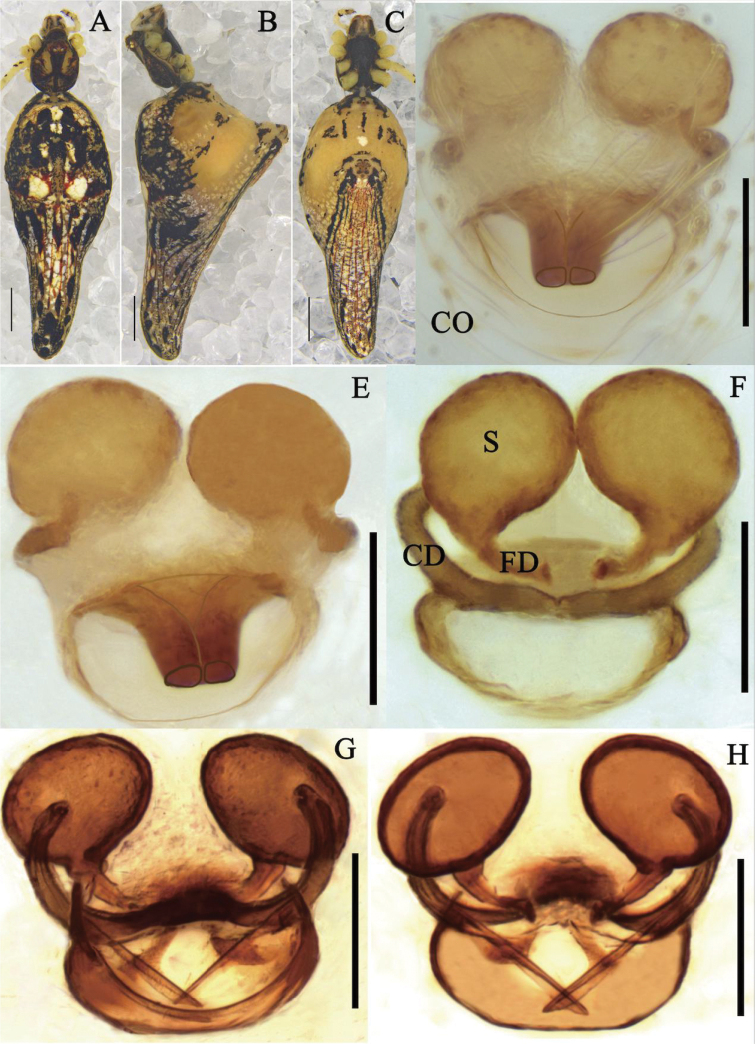
*Meotipapseudopicturata* sp. nov. **A–C** female habitus (flattened black spines on the abdomen and legs were broken off during taking photographs) (**A** dorsal **B** prolateral **C** ventral). **D–H** epigynum (**D** ventral, on the body **E, F** in the alcohol **E** ventral **F** dorsal **G, H** in gum arabic **G** ventral **H** dorsal). Scale bars: 1 mm **(A–C)**; 0.1 mm **(D–H)**. Abbreviations: CO = copulatory opening, CD = copulatory duct, FD = fertilization duct, S = spermathecae.

*Meotipapseudopicturata* sp. nov. is similar to *M.picturata* (see Murthappa et al. 2017: figs 1A–J, 2A–F, 4A–D) and *M.sahyadri* (see Kulkarni et al. 2017: figs 1–38) in having a deep round atrium, and having the copulatory ducts openings in the atrium (Fig. 7E). It can be distinguished from the latter two by the following combination of characters: (1) The tip of rod-shaped projection is narrower than its basal part in this new species, but wide in *M.picturata* (see Murthappa et al. 2017: fig. 4C) and *M.sahyadri* (see Kulkarni et al. 2017: fig. 21) ; (2) the ends of the copulatory ducts are curved and enter the spermathecae ventrally in *M.pseudopicturata* sp. nov. (Fig. 7F), and laterally in *M.picturata* (see Murthappa et al. 2017: figs 1J, 4D) and *M.sahyadri* (see Kulkarni et al. 2017: fig. 18); (3) fertilization ducts relatively short, proximal part confronting without curve in *M.pseudopicturata* sp. nov. (Fig. 7H), but relatively long, proximal part confronting with an apical curve in *M.picturata* (see Murthappa et al. 2017: fig. 4D) and *M.sahyadri* (see Kulkarni et al. 2017: fig. 18). In addition, *M.pseudopicturata* sp. nov. can be distinguished from other *Meotipa* species by the long and triangular opisthosoma which extends beyond spinnerets ~ 3/4 of total abdomen length, with blunt end curved ventrally.

##### Description.

**Female (holotype).** Total length 3.31; Prosoma length 1.08, width (at middle) 1.11, height (at middle) 0.69; Opisthosoma length 2.26, width (at middle) 0.91, height (at middle) 1.83; Eye diameters: ALE 0.07, AME 0.08, PLE 0.08, PME 0.07; Eye interdistances: AME–AME 0.05, ALE–ALE 0.10, PLE–ALE contiguous, PLE–PLE 0.26, PME–PME 0.10, PME–PLE 0.08, AME–ALE 0.03; Clypeus height (at middle) 0.43, width (at middle) 0.15; Measurements of legs: Leg I (right) 12.44 [4.23, 0.54, 2.58, 4.20, 0,89], II (right) 8.11 [2.73, 0.44, 1.65, 2.55, 0.74], III (right) 4.7 [1.68, 0.31, 0.99, 1.28, 0.44], IV (right) 10.91 [4.06, 0.38, 2.42, 3.44, 0.61]. Prosoma anteriorly and posteriorly truncated, medially flat, glabrous; cephalic area elevated. Carapace with black stripes, medially radiating streaks; fovea broad, smooth with distinct depression and irregular ridges (Fig. 7A). All eyes nearly uniform in size. Sternum black, heart shaped (Fig. 7C). Labium contiguous with the sternum, short with small hairs. Chelicerae vertical, black with red fangs (Fig. 7A–C). Femur, patella, and tibia with lanceolate spines distally; tibia with reddish black spot distally; leg segments except tibia with short, fine, dense hairs; femur IV with a row of lanceolate spines distoventrally; metatarsus I blackish distally. Leg formula 1423. Pedipalp short relative to its body size; tibia dorsally with lanceolate spines, long proximally, distally small with swelling. Opisthosoma triangular, very broad in lateral view, its dorsum provided with long alternate red and black stripes and white spots, caudal region knobbed extending downwardly towards spinnerets, provided with lanceolate spines. Venter transparent, some small black and white patches posteriorly (Fig. 7C). Atrium broad, with a rod-shaped projection apically (Fig. 7D); copulatory openings depressed, contiguous, located on the end of rod-shaped projection (Fig. 7E); copulatory ducts moderately long, tube curved extremely entering into ventral view of spermatheca (Fig. 7F); spermathecae oval-shaped, separated; fertilization duct thick, originating from basal spermathecae (Fig. 7H).

**Male.** Unknown.

##### Distribution.

China (Yunnan) (Fig. 10).

#### ﻿ Meotipa striata sp. nov.

Taxon classificationAnimaliaAraneaeTheridiidae

24EE6273-EF0C-5706-A8AF-0FBE2A31CF53

http://zoobank.org/1FA8D560-A36E-4A56-80F8-F7662B2BF3CB

Figures 8A–H , 10

##### Type material.

***Holotype*** female (CBEE). **Yunnan Province**: Mengla County, Wangtianshu Scenic Area (21.62°N, 101.58°E, 680 m), 15 November 2019, J. Chen, J. Liu, Z.C. Li & B. Liang leg.

##### Etymology.

The species epithet refers to the black and yellow stripes on the abdomen of the specimen.

##### Diagnosis.

**Figure 8.**
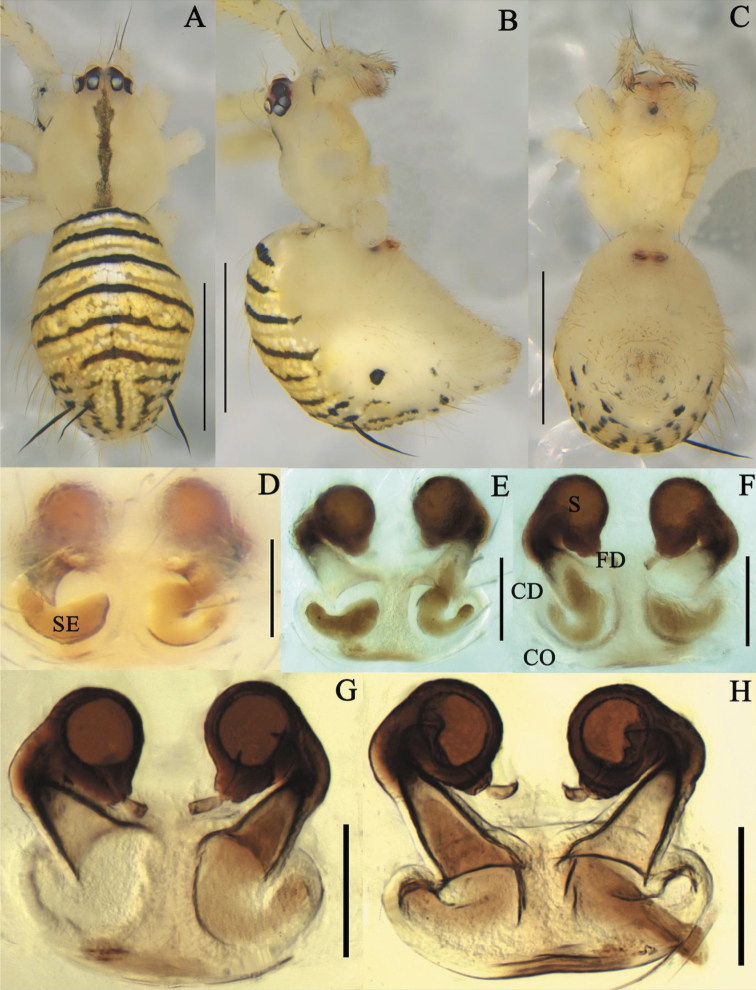
*Meotipastriata* sp. nov. **A–C** female habitus (some flattened black spines on the abdomen and legs were broken off during taking photographs) (**A** dorsal **B** prolateral **C** ventral). **D–H** epigynum (**D** ventral, on the body **E, F** in the alcohol **E** ventral **F** dorsal **G, H** in gum arabic **G** ventral **H** dorsal). Scale bars: 1 mm **(A–C)**; 0.1 mm **(D–H)**. Abbreviations: CO = copulatory opening, CD = copulatory duct, FD = fertilization duct, S = spermathecae, SE = stuck emboli.

The new species is similar to *M.spiniventris* (Figs 4A–H, 5A–H) and *M.capacifaba* (see Li et al. 2020: figs 1A–J, 2A–E, 3A–E) in sharing characters such as the raised eyes, a marking in the middle of carapace, and two conspicuous copulatory openings. It can be distinguished from the two species by the following characters: (1) the atrium is divided into two circular openings by the epigynal septum in *M.capacifaba* (see Li et al. 2020: figs 2A, B) and *M.spiniventris* (Fig. 4D–H), but not in *M.striata* sp. nov.; (2) the copulatory ducts gradually narrow from copulatory openings to spermathecae in *M.striata* sp. nov. (Fig. 8F) and *M.spiniventris* (Fig. 3F), but swell into sphere-shaped in *M.capacifaba* (see Li et al. 2020: figs 2A, B).

##### Description.

**Female (holotype).** Total length 2.41; Prosoma length 0.96, width (at middle) 0.83, height (at middle) 0.61; Opisthosoma length 1.4, width (at middle) 1.10, height (at middle) 1.50; Eye diameters: ALE 0.07, AME 0.08, PLE 0.08, PME 0.08; Eye interdistances: AME–AME 0.03, ALE–ALE 0.23, PLE–ALE contiguous, PLE–PLE 0.28, PME–PME 0.08, PME–PLE 0.06, AME–ALE 0.03; Clypeus height (at middle) 0.26, width (at middle) 0,14; Measurements of legs: Leg I (right) 9.30 [2.80, 0.45, 2.12, 3.01, 0.92], II (right) 5.85 [1.96, 0.36, 1.21, 1.51, 0.81], III (right) 4.85 [1.40, 0.22, 0.80, 1.39, 0.50], IV (right) 6.86 [2.50, 0.40, 1.42, 1.87, 0.67]. Carapace rather ovate with bar-shaped longitudinal narrow fovea; cephalic area relatively short and narrow. All eyes uniform in size, strongly recurved; dark AME surrounded by red-brown rings; PME pearly white surrounded by black rings (Fig. 8A). Sternum yellow, subtle heart-shaped, lateral margins slightly indented (Fig. 8C). Labium contiguous with the sternum, white, approximately triangular in shape. Chelicera vertical, white with black fang (Fig. 8B). Legs yellowish white with small hairs and bearing a few dark short spines; coxae and femora white with pale black spots; the ends of metatarsi with pale brown band (Fig. 8B). Leg formula 1423. Pedipalp white, short, single-clawed, with many short hairs. Opisthosoma oval, provided with yellowish setae. Abdomen with black patch and snowy white patches medially, dorsally with four lanceolate spines; spinnerets oriented downwards (Fig. 8A). Copulatory openings large, separated from each other, with mating plugs (Fig. 8D); copulatory ducts gradually narrow from copulatory opening to spermathecae and bend, vertical at lateral sides of spermathecae (Fig. 8F); spermathecae spherical (Fig. 8G); fertilization ducts located on the basal of spermathecae, facing each other (Fig. 8H).

**Figure 9.**
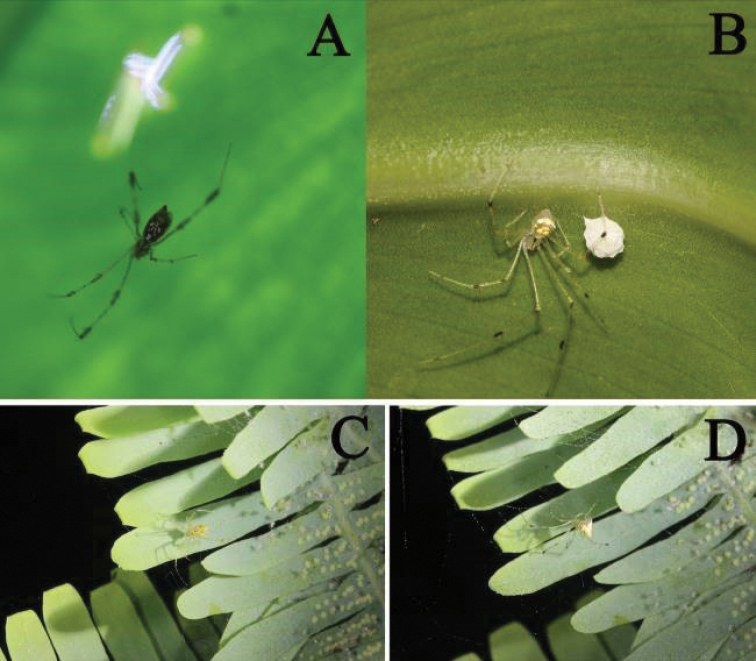
Field photographs **A***M.picturata* female on its web **B***M.spiniventris* female with egg sac **C, D***M.pulcherrima* female with its spiderlings. Photographs by Zhongwei Deng in Yunnan Province.

**Male.** Unknown.

##### Distribution.

**Figure 10.**
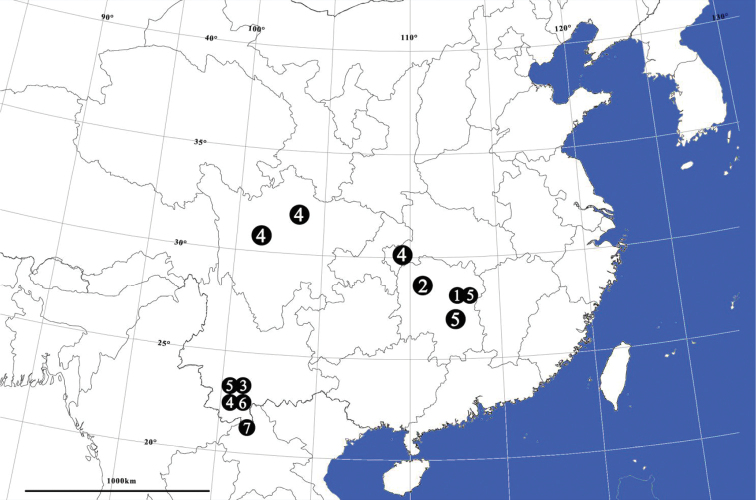
Map with sampling localities for *Meotipa* species from China: 1 *M.argyrodiformis* 2 *M.pulcherrima* 3 *M.picturata* 4 *M.spiniventris* 5 *M.vesiculosa* 6 *M.pseudopicturata* sp. nov. 7 *M.striata* sp. nov..

China (Yunnan) (Fig. 10).

## ﻿Discussion

*Meotipa* Simon, 1895 is a relatively small genus of the family Theridiidae, totaling 20 species globally including two new species reported here. Most species are distributed in East Asia (eleven species in China, three species in Japan and Korea), Southeast Asia (eight species in Brunei, Indonesia, Laos, Malaysia, Myanmar, Thailand, and Vietnam), and South Asia (five species in India). Only *M.pulcherrima* is found outside Asia: it is widely distributed in tropical Africa and is presumed to have been introduced to Papua New Guinea, China, Korea, Japan, and the Pacific islands, and the Americas, including its type locality in Brazil (see Note under *M.pulcherrima*). *Meotipa* spiders prefer to inhabit the underside of leaves typically decorated by lichens and fungi. Against this background, the black, white, and red patterns on the body and the brushes of black scale-like spines on legs and abdomen blur their outline and enhance their disguise (Deeleman-Reinhold 2009). Therefore, these long-legged spiny theridiids are difficult to find and collect in the wild. Given the broad distribution of *Meotipa* in Asia spanning many poorly sampled areas where taxonomic expertise is lacking, it is likely that many species await discovery and thus further taxonomical work focused on this genus is needed.

It is difficult to speculate on *Meotipa* interrelationships without a phylogeny, but some morphological observation may serve to indicate certain relationships: 1. Abdomen bearing two pairs of humps on dorso-lateral sides is only present in *M.impatiens*, *M.ultapani*, and *M.vesiculosa*; 2. A rod-shaped projection in the epigynum is found in *M.picturataM.sahyadri*, and *M.pseudopicturata* sp. nov.; 3. The copulatory ducts are spherical in *M.capacifaba* and *M.pulcherrima*, but distinctly curved in *M.capacifaba* and *M.pulcherrima*; 4. The conductor is spoon-shaped in *M.argyrodiformis*, *M.menglun*, *M.picturata*, *M.sahyadri*, and *M.vesiculosa*. These characters have minimum overlap (*M.vesiculosa* may belong to two of the groups) and might suggest the following species groups and relationships: (*M.impatiens*, *M.ultapani*, and *M.vesiculosa*),((*M.picturata*, *M.sahyadri*, and *M.pseudopicturata*) *M.argyrodiformis*)), (*M.capacifaba* and *M.pulcherrima*). However, whether these characters actually reflect relationships remains to be seen upon the systematic collection of phylogenetic data. In addition, we must consider that the two new species (only female) covered in the current paper were collected from same region (Mengla County, Xishuangbanna Dai Autonomous Prefecture) with three known species (only males of *M.luoqiae*, *M.menglun*, *M.zhengguoi*) recently reported (Lin et al. 2021), but we are sure that they do not match with each other based on the following reasons: 1. the habitus of our new species (*M.pseudopicturata* sp. nov. and *M.striata* sp. nov.) are significantly different from the males of the three known species (Figs 7A–C, 8A–C); 2. the male of *M.pseudopicturata* sp. nov. should be similar to *M.picturata* and *M.sahyadri* according to our above hypothesis about *Meotipa* interrelationships, but none of these three species is similar; 3. the female of *M.striata* sp. nov. is similar to *M.spiniventris* (Figs 4A–H, 5A–H) and *M.capacifaba* (see Li et al. 2020: figs 1A–J, 2A–E, 3A–E) in having two conspicuous copulatory openings; therefore, we speculate that its male should be similar to *M.spiniventris* (Figs 4A–H, 5A–H) and *M.capacifaba* in having a straight embolus covered completely by conductor, but the embolus is not covered by the conductor in these three species (*M.luoqiae*, *M.menglun*, and *M.zhengguoi*; see Lin et al. 2021: figs 47–52).

*Meotipa* clearly belongs to the subfamily Theridiinae based both on molecular phylogenetic data (Liu et al. 2016), and clear morphological synapomorphies, in particular the complete absence of the colulus and the hooded paracymbium (Agnarsson 2004a). The exact position of *Meotipa* within Theridiinae is less certain: Deeleman-Reinhold (2009) resurrected *Meotipa* from *Chrysso* mainly based on the presence of flattened black spines on the abdomen and/or legs, and the cutinized conductor in the male palp. However, the genus *Chrysso* seems to be polyphyletic (Liu et al. 2016). Deeleman-Reinhold (2009) speculated that the *Chrysso* species distributed in Southeast Asia are more closely related to *Theridion* and *Achaearanea* than they are to the type species *Chryssoalbomaculata* from the Americas (Deeleman-Reinhold, 2009). We do not see any evidence of the close relationship between *Theridion* and *Meotipa* (but note that Agnarsson et al. (2007b) included an undetermined *Meotipa* species from Malaysia but its placement as sister to *Theridion* was poorly supported). However, the type of *Achaearanea*, *A.trapezoidalis*, shares certain characteristics such as abdomen shape with many species of *Chrysso*. The study of Liu et al. (2016) included one unnamed *Meotipa* species, placing it on a long branch as sister to two *Yunohamella* species: *Y.lyricus* (Walckenaer, 1841) and *Y.palmgreni* (Marusik & Tsellarius, 1986) according to recent research (Marusik and Logunov, 2017). We infer that *Meotipa* may instead belong to the “*Chrysso* clade” of Arnedo et al. (2007), given the similarities between *Meotipa* and *Chrysso*. Such speculations need to be tested through innovative phylogenetic analyses aimed to anchor *Meotipa* to the theridiid tree of life.

## Supplementary Material

XML Treatment for Meotipa

XML Treatment for Meotipa argyrodiformis

XML Treatment for Meotipa pulcherrima

XML Treatment for Meotipa picturata

XML Treatment for Meotipa spiniventris

XML Treatment for Meotipa vesiculosa

XML Treatment for Meotipa pseudopicturata

XML Treatment for Meotipa striata
